# Experimental ex-vivo performance study comparing a novel, pulsed thulium solid-state laser, chopped thulium fibre laser, low and high-power holmium:YAG laser for endoscopic enucleation of the prostate

**DOI:** 10.1007/s00345-021-03825-z

**Published:** 2021-09-03

**Authors:** Mehmet Yilmaz, Julia Esser, Lea Kraft, Ralf Petzold, August Sigle, Christian Gratzke, Rodrigo Suarez-Ibarrola, Arkadiusz Miernik

**Affiliations:** Faculty of Medicine, Department of Urology, University of Freiburg-Medical Centre, Hugstetter Str. 55, 79106 Freiburg, Germany

**Keywords:** Benign prostatic hyperplasia, Holmium laser, Pulsed thulium laser, Thulium fibre laser, Transurethral endoscopic enucleation of the prostate

## Abstract

**Purpose:**

The aim of this study was to compare the enucleation performances of four different types of laser devices in an ex-vivo experiment: a novel, pulsed Tm:YAG solid-state laser evaluation model (p-Tm:YAG), chopped thulium fibre laser (TFL), low-power Ho:YAG laser (LP-Ho:YAG), and a high-power Ho:YAG laser (HP-Ho:YAG).

**Methods:**

Our primary aim was to endoscopically separate the fascial layers of a porcine belly using laser fibres within a time period of 60 s. The size of a “tissue pocket” was assessed numerically. The enucleation characteristics reflecting the surgeon’s experience were evaluated via the NASA Task Load Index (TLX) questionnaire and a questionnaire based on Likert scale.

**Results:**

HP-Ho:YAG achieved with the available laser settings the largest overall “tissue pocket” (31.5 cm^2^) followed by p-Tm:YAG (15 cm^2^), TFL (12 cm^2^), and LP-Ho:YAG (6 cm^2^). The coagulation performances of p-Tm:YAG and TFL were rated the best. In the performance evaluation by the Likert questionnaire, HP-Ho:YAG (average score of 4.06) was rated highest, followed by p-Tm:YAG (3.94), TFL (3.38), and LP-Ho:YAG (3.25). The evaluation of the NASA-TLX performance questionnaire revealed average scores for HP-Ho:YAG, LP-Ho:YAG, TFL and p-Tm:YAG of 4.38, 4.09, 3.92 and 3.90, respectively.

**Conclusion:**

We are the first to compare different laser devices and settings in an ex-vivo study. We found that the surgeons were most satisfied with the HP-Ho:YAG laser device, followed by the p-Tm:YAG. These findings could be highly relevant for future research and for the practical utilisation of laser systems in endourology.

**Supplementary Information:**

The online version contains supplementary material available at 10.1007/s00345-021-03825-z.

## Introduction

Due to the progress made in laser technologies, endoscopic enucleation of the prostate (EEP) has become increasingly popular in the surgical treatment of benign prostate hyperplasia (BPH) [1]. Holmium:YAG lasers, thulium:YAG lasers, lithium triborate (LBO) crystal lasers, potassium-titanyl-phosphate (KTP) lasers (GreenLight laser system) and diode lasers have been the most often employed laser devices in the surgical treatment of lower urinary tract symptoms (LUTS) caused by BPH [2]. The chopped thulium fibre laser (TFL) and pulsed Tm:YAG solid-state laser (p-Tm:YAG) evaluation model used in our study are recent innovations that have the potential to play a larger role in the clinical practice of laser energy-based enucleation; they may even replace currently standard laser systems [3, 4]. During the enucleation process, prostate adenoma is detached from the surgical pseudocapsule using laser energy and usually blunt dissection. Tissue damage is thus intentionally induced by laser energy. The laser energy absorbed by water interferes with the surroundings via thermal or mechanical interactions [5]. This process should be easy to control by well-judged energy dosage and application, resulting in negligible collateral effects. Continuous-wave and pulsed lasers have a different biophysical impact on tissue which can cause damage to both the tissue itself and the medical devices used during surgery [1]. In addition to the surgical technique, enucleation safety and efficiency could therefore be substantially dependent on the laser system and its setting. It is thus essential to be aware of the effects of laser-tissue interaction and select optimum settings to increase the efficacy of enucleation and prevent complications.

A notable number of studies have focussed on clinical parameters concerning different types of laser devices typically used to enucleate the prostate [6–9]. However, there have been few investigations assessing different lasers, their genuine biophysical impact, and how they interact with tissue. The aim of this study was to compare the enucleation performance of different commercially available laser devices, a chopped TFL, a low-power holmium:YAG laser (LP-Ho:YAG) and a high-power holmium:YAG laser (HP-Ho:YAG), against the new evaluation model pulsed thulium laser technology. Prostate enucleation was simulated by separating fascial layers of porcine belly tissue. To the best of our knowledge, there have been no investigations to date demonstrating the enucleation performance of four different laser types in the same ex-vivo experiment.

## Materials and methods

Four different types of laser devices were used to investigate the enucleation performance in ex-vivo experiments simulating prostate enucleation. Table 1 (Online Resource 1) shows the basic characteristics of each laser type.

Our experimental setup is illustrated in Fig. 1 (Online Resource 2). All experiments were conducted using a 26-Fr continuous-flow laser resectoscope (Richard Wolf, Knittlingen, Germany). In our setup, we chose to employ a porcine belly. A porcine belly’s anterior abdominal wall has intermuscular fascial layers which possess texture closely resembling that of the human prostate’s surgical capsule. The porcine belly, completely covered by saline solution, was attached to the bottom of a plastic box by a surgical retractor system using fastening hooks. The resectoscope entered the porcine belly through a hole in front of the box, exactly as big as the resectoscope shaft to simulate the resectoscope’s restricted freedom of movement within the male lower urinary tract (Online Resource 2). Each laser fibre, as listed in Table 1, was inserted through the endoscope. Each repetition was recorded by a live camera image transmission displayed on the monitor. For the image and light transmission, a camera head (ENDOCAM Logic HD, Richard Wolf GmbH, Knittlingen, Germany) and a light source (ENDOLIGHT LED Blue, Richard Wolf GmbH, Knittlingen, Germany) were used.

We conducted a total of 18 experiments, each investigating the enucleation performance of one specific laser device and setting. The surgeon’s aim was to separate intermuscular fascial layers of a porcine belly’s anterior abdominal wall using laser energy and mechanical retraction, thereby dissecting as much tissue as possible within 60 s. Due to that, template weighting was not possible. There were no restrictions regarding the surgical technique. The surgeon had carried out over 1000 transurethral prostate procedures, of which over 650 were EEPs. He was instructed to adapt the speed and his dissection technique to the current intraoperative conditions trying to achieve best performance possible. From the biophysical perspective, these restrictions cause that no direct comparison between systems is possible. Due to fundamental differences in the laser technology and pulse generation, it was impossible to ensure equal pulse durations on all the laser devices. We intended to explore specific and maximum settings of the devices to better understand their potentially unique properties for EEP.

The enucleation performance of each laser was evaluated by taking four different approaches to make our results more congruent. The first method was a numerical measurement of “the tissue pocket” created during the process. Secondly, the surgeon rated the enucleation performance applying a 5-point Likert scale (Table 2—Online Resource 1) (1: “strongly disagree”, 2: “disagree”, 3: “neutral”, 4: “agree”, 5: “strongly agree”). Among these, precision and cutting ability were the two subjective items, while speed and coagulation were the two objective items.

The third assessment method was the National Aeronautics and Space Administration Task Load Index (NASA TLX) questionnaire after each experiment. This standardized questionnaire enables the performance assessment through six items (Mental demand, Physical demand, Temporal demand, Performance, Effort and Frustration). To be able to compare its results with the Likert scale’s, the rating system was rescaled in accordance with the highest achievable score (5 points): “very low” (1) or “perfect” (5). We evaluated the questionnaire by determining the statistical means of each item belonging to each laser device and rescaled them accordingly.

In the last method we applied, an independent, non-urologist observer who is an engineer specialising in medical technologies and experienced in endourological laser applications assessed the coagulation effect using the 5-point Likert scale with the help of recorded videos of the experiments.

The completed Likert and NASA-TLX questionnaires on each laser device were assessed for their standard distribution via the Shapiro–Wilk test. Homogeneity of variances was examined using Levene’s Test. To evaluate whether the observed differences were statistically significant, we conducted Kruskal–Wallis *H* test followed by a Dunn’s test (based on the Bonferroni correction principle) as post-hoc analysis. The significance level for all tests was 0.05.

## Results

### Tissue pocket size

Only pre-set laser settings could be tested with the evaluated laser systems, which are summarized in Table 3 (Online Resource 1). After separating the fascial layers using the laser for just 60 s, the tissue pocket size was calculated using the formula of the ellipse area: Area = π.*a*.*b*. The largest overall pocket size (31.5 cm^2^) was achieved with the HP-Ho:YAG laser (4.5 J, 22.3 Hz, 100 W, 0.15 ms). An increase of the pulse duration or a lower average power of this laser system, reduced the formed pocket size. The LP-Ho:YAG formed the largest pocket size (6 cm^2^) at 3.5 J, 10 Hz, 35 W and a pulse duration of 0.45 ms. Reducing the pulse energy also decreases the obtained pocket sizes. 12 cm^2^ were the largest pocket size achieved with the TFL system at 4 J, 10 Hz, 40 W, and 8 ms pulse duration. By adjusting the pulse energy and pulse duration to smaller or larger values using similar average power, pocket sizes no larger than half were formed. The high frequency setting (0.2 J, 200 Hz, 40 W, 0.4 ms) of the TFL produced the smallest overall pocket size (0.25 cm^2^). The maximum pocket size formed by the p-Tm:YAG laser system was 15 cm^2^ at 3 J, 25 Hz, 75 W, and 0.86 ms pulse duration. Only smaller pocket sizes were achieved for the other settings tested with lower pulse energies or higher frequencies.

Figure 2 (Online Resource 3) illustrates four differently-sized tissue pockets formed by different laser devices at the same pulse energy and frequency (3 J, 10 Hz) but at dissimilar pulse durations. The pocket sizes formed by LP-Ho:YAG, p-Tm:YAG, HP-Ho:YAG and TFL were 2 cm^2^, 3 cm^2^, 4.5 cm^2^ and 5.25 cm^2^, respectively.

### Likert scale and NASA-TLX evaluations

The individual performances of the tested laser systems in the Likert and NASA-TLX questionnaires are shown as net diagrams in Fig. 3 (A and B) (Online Resource 4). For the Likert questionnaire, the HP-Ho:YAG rated best in enucleation speed and cutting ability whereas the p-Tm:YAG and TFL showed better coagulation behaviour. In the evaluation of NASA-TLX questionnaire, the HP-Ho:YAG laser scored highest in all areas except temporal demands. The LP-Ho:YAG reached highest in temporal demands and were rated as second least frustrating. The p-Tm:YAG achieved comparable results to the TFL system.

Figure 3 (C) (Online Resource 4) shows the average Likert and NASA-TLX questionnaires scoring for each laser device across all experiments. The average Likert questionnaire scores were 3.25, 3.38, 3.94 and 4.06 for LP-Ho:YAG, TFL, p-Tm:YAG and HP-Ho:YAG, respectively. The surgeon found the HP-Ho:YAG to be the most satisfactory among all lasers, with a Likert score of 4.06 out of 5. The LP-Ho:YAG (*W* = 0.80, *p* = 0.002), TFL (*W* = 0.84, *p* = 0.001), p-Tm:YAG (*W* = 0.81, *p* = 0.003), and HP-Ho:YAG (*W* = 0.80, *p* = 0.002) scores were not normally distributed. Equal variances were assumed (*F* = 0.53, *p* = 0.67). The result of the Kruskal–Wallis *H* test showed a major difference in the scoring of the laser devices (*H* = 13.1, *p* = 0.004). Post-hoc Dunn’s test revealed a significant difference only between the HP-Ho:YAG and both the LP-Ho:YAG (*p* = 0.02) and the TFL (*p* = 0.04).

Each laser device’s average NASA-TLX questionnaire scores for HP-Ho:YAG, LP-Ho:YAG, TFL and p-Tm:YAG were 4.38, 4.09, 3.92 and 3.90, respectively across all experiments (Fig. 3C). Consistent with the Likert questionnaire results, HP-Ho:YAG proved most satisfactory for the surgeon. LP-Ho:YAG (*W* = 0.88, *p* = 0.007), TFL (*W* = 0.84, *p* = 0.0001), p-Tm:YAG (*W* = 0.85, *p* = 0.002), and HP-Ho:YAG (W = 0.80, p = 0.0003) scores were not normally distributed. Equal variances were not assumed (*F* = 2.87, *p* = 0.04). The result of the Kruskal–Wallis *H* test indicated a significant difference in the scoring of the laser devices (*H* = 16.5, *p* = 0.0009). Post-hoc Dunn’s test revealed that only the HP-Ho:YAG differed significantly from both the p-Tm:YAG (*p* = 0.009) and TFL (*p* = 0.0007).

### Coagulation performance

Figure 3D (Online Resource 4) shows the average coagulation-performance scores assessed by an independent, non-urologist observer after watching the experiment videos. p-Tm:YAG earned the highest average score (4.3), followed by TFL (3.5) and LP-Ho:YAG (3.0). Assigned a value of 2.5, the HP-Ho:YAG was the least satisfactory.

## Discussion

Due to the widespread practice of laser prostate enucleation, many studies focussed on determining the most effective and safest type of laser for enucleating the prostate. In the present study, we examined various enucleation characteristics considering four different laser systems, whether established or “newcomers” for EEP. Our results reveal that the HP-Ho:YAG formed the overall the largest tissue pocket under experimental ex-vivo conditions, followed by the p-Tm:YAG, TFL and LP-Ho:YAG. Since only pre-set parameters were possible, a comparison of the laser systems used is only possible to a limited extent. To the best of our knowledge, ours is the first study to investigate and compare the enucleation performance of four different types of laser devices in the same ex-vivo experiment.

To comprehensively evaluate the enucleation efficiency, we used the Likert scale and NASA TLX to assess the laser devices enucleation performance subjectively. It is noteworthy that the Likert scale was intendedly created for the study. In contrary, the NASA index is a general assessment tool for technology of any kind. In summary, this might give a solid outline regarding the clinical utility but differ in scoring. According to the Likert scale, the HP-Ho:YAG proved overall to be the most satisfactory laser device for the surgeon. It differed significantly from both the LP-Ho:YAG (*p* = 0.02) and TFL (*p* = 0.04). According to the NASA TLX, the HP-Ho:YAG also scored highest and differed significantly from both the p-Tm:YAG (*p* = 0.009) and TFL (*p* = 0.0007). The surgeon’s satisfaction with HP-Ho:YAG laser might be attributable to his familiarity using the holmium laser. In contrast to the abovementioned results, the p-Tm:YAG’s coagulation performance was rated highest by the independent observer after watching the live videos, followed by TFL and LP-Ho:YAG; the HP-Ho:YAG was rated the lowest. This should be although interpreted with caution because in an ex-vivo model coagulation cannot be compared to the genuine intraoperative situation. Since the enucleation experiment was performed by the same surgeon, who also completed the questionnaires, discrepancy between his findings and those of the independent observer is to be anticipated.

Lerner et al. reviewed the most widely practiced laser enucleation approaches along with key characteristics of each wavelength, but they left open the question “What approach and/or energy source is best?” [1]. We show that the HP-Ho:YAG at a setting of 4.5 J, 22.3 Hz and a low optical-pulse duration of 0.15 ms was associated with the highest dissection efficiency. However, there is no consensus among surgeons performing EEP as to which setting is best, as other intraoperative parameters (e.g. blunt dissection) must also be considered. We conclude overall that high frequencies (> 30 Hz) are inferior for rapid dissecting. Both dissection efficiency and the laser fibre’s controllability seem to diminish as the pulse frequency rises.

Holmium laser ablates tissue effectively and is safe thanks to its pulsed nature and 0.4 mm penetration depth [1, 2]. Numerous authors have focussed on the clinical outcome of laser procedures and device settings [4, 8, 10, 11]. In a randomised controlled trial, Elshal et al. showed that the LP-HoLEP (2 J/25 Hz) and HP-HoLEP (2 J/50 Hz) are comparable in terms of enucleation efficiency regardless of the surgeon’s experience (1.42 ± 0.6 g/min vs. 1.47 ± 0.6 g/min, respectively) [11]. Similarly, while performing HoLEP, Shah et al. detected no significant difference between medium power (2 J/25–30 Hz) and high power (2 J/50 Hz) laser settings in functional outcome and complications [9]. On the other hand, Gazel et al. compared two different low-power settings during enucleation and haemostasis. They reported that enucleation efficiency was higher when applying 37.5 W than 20 W when employing a HoLEP (p = 0.032) [12]. We found that a low power (3 J/10 Hz) laser setting for the HP-Ho:YAG resulted in half of the efficiency (cm^2^/min) at a medium power (3 J/25 Hz) laser setting. We also observed that in general, high-power systems with high single-pulse energy, short pulses, and medium frequency deliver the best dissection outcome.

Thulium laser technology has improved enormously in the last decade. Until now, most dominant Tm:YAG lasers have been high-power continuous wave (CW) lasers (2013 nm). Besides that, TFL is on the market (1940 nm). A novel pulsed thulium solid-state laser was only recently developed (2013 nm), so there have been no comparative studies with the p-Tm:YAG up to the time of our study.

The efficiency and safety of (CW) Tm:YAG application have been investigated [10, 13, 14]. Yet experimental studies performed on real tissue are scarce. The first ex-vivo study investigating the (CW) thulium laser’s effect on an isolated blood-perfused porcine kidney was conducted by Wendt-Nordhal et al. They showed that a (CW) thulium laser (2013 nm) at a power setting of 70 W has a higher tissue-ablation rate than a 80 W KTP laser [15]. In another ex-vivo study employing an isolated blood-perfused porcine kidney, Bach et al. demonstrated that 120 W (CW) Tm:YAG is associated with higher ablation rates than a 70 W setting [16]. Pirola et al. detected negligible clinical differences between a high-power Ho:YAG laser (100 W) and high-power (CW) Tm:YAG laser (110 W) in efficacy and safety [8]. Two meta-analyses revealed that (CW) ThuLEP showed higher enucleation efficacy than HoLEP [17, 18]. However, their results are not comparable with our data, hence no pulsed thulium laser could have been investigated at that time. However, our results show that the p-Tm:YAG is also more efficient at ablating tissue than the HP-Ho:YAG at the same laser settings in terms of single-pulse energy and frequency (3 J/25 Hz/75 W).

TFL was developed recently and is used to endoscopically enucleate the prostate (ThuFLEP) [4]. It exhibits better energy absorption in water and thus shallower penetration depth than Ho:YAG and Tm:YAG [19]. In an ex-vivo study on canine prostate, Fried et al. showed that 40 W TFL is capable to attain an ablation rate at 0.2 g/min. [20]. They concluded that this rate would not have been rapid enough in genuine BPH surgery. Another ex-vivo study by the same group found that utilising a 110 W TFL tissue-ablation rate increased by four-fold 40 W [21]. Enikeev et al. reported no significant difference between HoLEP and ThuFLEP in terms of enucleation rates (1.9 ± 0.74 g/min vs 1.9 ± 0.69 g/min; *p* = 0.217) [22]. However, in the present study, we found that the TFL was on average less efficient (cm^2^/min) than the HP-Ho:YAG, but more efficient than the LP-Ho:YAG.

Our study has certain limitations. First, only one laser setting (3 J, 10 Hz) could be applied on all the laser devices we examined. Therefore, we had to focus on laser-specific settings important for enucleation that are displayed in Table 3 (Online Resourse 1). The laser settings therefore cannot be specifically compared as such. This is due to the laser technology itself and the design of the light emitters. Secondly, we studied an ex-vivo non-perfused model. Again, we are aware that this thermal effect is not readily transferable to human organs such as the prostate in terms of its coagulation ability. Our examinations were carried out by a single surgeon highly experienced in endourology. There is no realistic biological model for ex-vivo experimental EEP research. That is why we decided to work on the model proposed above. Successful EEP requires a thermal energy source (beside mechanical interaction knows as blunt dissection). Due to our study design investigating four laser systems at different settings, operation time was limited to 60 s. This may have had an important impact on our results, as we could only analyse a very specific part of the procedure. In addition, a part of our evaluation could only be done by relying on questionnaires. Due to the participation of only one surgeon, the bias through his personal experience might be significant. Experiments engaging different surgeons would make such results more objective.

We believe that comparing several different lasers at the same experimental setting is more useful than comparing a single laser at various settings. By creating an experimental setup closely resembling the clinical conditions of EEP, we believe our results could contribute valuable knowledge to facilitate improved utilisation of different laser energy sources in endourology. More clinical data is needed to prove these experimental findings. We believe that this work will facilitate research on lasers for EEP and may help surgeons decide when choosing an energy source for enucleation. The main objective is to further improve functional outcomes and quality of life among patients with BPH suffering from LUTS undergoing EEP.

## Conclusions

Different laser systems are being increasingly used to surgically treat LUTS in BPH patients. We explored four laser systems at different settings suitable for EEP under standardised experimental ex-vivo conditions. Our results deepen our knowledge about the targeted deployment of lasers in endourology. As it delivered the best enucleation performance, the HP-Ho:YAG proved to be the most satisfactory laser device for the surgeon, followed by the p-Tm:YAG. However, as this research project was experimental in nature, its findings’ clinical relevance is probably limited and will have to be confirmed, particularly by clinical investigations.

## Supplementary Information

Below is the link to the electronic supplementary material.

Supplementary file1 Online Resource 1 Table 1: Basic characteristics of the laser devices according to manufacturer’s specifications with λ: wavelength in nanometers, α: (approximate) water absorption coefficient. Esp: energy of an optical pulse in Joule, τ: duration of an optical pulse in milliseconds, f: pulse repetition rate in Hertz, P: (maximum) average power in Watt. Table 2: Two objective and two subjective parameters evaluating the enucleation performance by the surgeon using 5-point Likert scale. Table 3: Formed pocket sizes in square centimetres for each laser setting and laser device. E_sp_: energy of an optical pulse in Joule, f: pulse frequency in Hertz, τ: duration of an optical pulse in milliseconds, P: average power in Watt. (PDF 344 KB)

Supplementary file2 Online Resource 2 Legend. Fig.1 Experimental setup: A) surgeon’s hands holding the endoscope, B) endoscope, C) and D) inflow and outflow of irrigation, E) box, F) porcine belly, G) image and light transmission system of endoscope, H) endoscopic image of the porcine belly, I) TFL, J) p-Tm:YAG, K) collagen fibres, L) laser fibre tip. (PDF 224 KB)

Supplementary file3 Online Resource 3 Legend. Fig.2 Tissue pockets created by different laser devices applying the same laser setting (3 J, 10 Hz): A): LP-Ho:YAG (2 cm²), B): TFL (5.25 cm²), C): HP-Ho:YAG (4.5 cm²), D): p-Tm:YAG (3 cm²) (PDF 101 KB)

Supplementary file4 Online Resource 4 Legend Fig.3 A and B: Laser devices’ mean scores on the Likert and NASA-TLX questionnaires illustrated as net diagram; C: Each laser device’s average score on the Likert and NASA-TLX questionnaires; D: Relation between average scores of coagulation performance and laser devices assessed by an independent, non-urologist observer. The red error bars indicate the standard deviation (SD) (PDF 138 KB)

## Data Availability

The raw data is with the corresponding author and can be provided on request.

## Code availability

Not applicable for this section.
