# Supplementary material for: Experimental ex-vivo performance study comparing a novel, pulsed thulium solid-state laser, chopped thulium fibre laser, low and high-power holmium:YAG laser for endoscopic enucleation of the prostate
Source: World J Urol. 2021 Sep 3;40(2):601–6. doi: 10.1007/s00345-021-03825-z (PMC8921029; doi:10.1007/s00345-021-03825-z)
Supplement: Supplementary file 1 — Supplementary file1 Online Resource 1 Table 1: Basic characteristics of the laser devices according to manufacturer’s specifications with λ: wavelength in nanometers, α: (approximate) water absorption coefficient. Esp: energy of an optical pulse in Joule, τ: duration of an optical pulse in milliseconds, f: pulse repetition rate in Hertz, P: (maximum) average power in Watt. Table 2: Two objective and two subjective parameters evaluating the enucleation performance by the surgeon using 5-point Likert scale. Table 3: Formed pocket sizes in square centimetres for each laser setting and laser device. Esp: energy of an optical pulse in Joule, f: pulse frequency in Hertz, τ: duration of an optical pulse in milliseconds, P: average power in Watt. (PDF 344 KB) [file 345_2021_3825_MOESM1_ESM.pdf]

Table 1: Basic characteristics of the laser devices according to manufacturer's specifications with λ: wavelength in nanometers, α: (approximate) water absorption coefficient, E_{SP}: energy of an optical pulse in Joule, τ: duration of an optical pulse in milliseconds, f: pulse repetition rate in Hertz, P: (maximum) average power in Watt.

**Parameter**	**Low-power Holmium solid-state laser**	**Pulsed Thulium solid-state laser**	**Thulium fiber laser**	**High-power Holmium solid-state laser**
**Operating mode**	Pulsed	Pulsed	Pulse generation by chopping CW laser beam	Pulsed
**Abbrevi-ation**	LP-Ho:YAG	p-Tm:YAG	TFL	HP-Ho:YAG
**Model**	MEDILAS® H SOLVO® 35	Evaluation model	YLR-2000-U	SPHINX®
**Manufac-turer**	Dornier MedTech Laser GmbH, Wessling, Germany		IPG Photonics © IRE-Polus, Fryazino, Russia	LISA laser products OHG, Katlenburg-Lindau, Germany
**Laser fiber**	Dornier SingleFlex® 400 µm (Dornier MedTech Laser GmbH)		400 µm (IPG Photonics © IRE-Polus)	RigiFib, 550 µm, (LISA laser products OHG, Katlenburg-Lindau, Germany)
**λ [nm]**	2080	2013	1940	2123
**α [mm^{-1}]**	3.2	5.9	14	3
**E_{SP} [J]**	0.1-3.5	0.1-3	0.025-6	0.5-4.5
**τ [ms]**	0.14-0.45	0.15-1	0.05-12	0.15-0.80
**f [Hz]**	3-25	5-200	6-1600	4-30
**P [W]**	35	120	40	100

Table 2: Two objective and two subjective parameters evaluating the enucleation performance by the surgeon using 5-point Likert scale.

**Likert item**	**Subjective Parameters**
Speed	I was able to dissect as much tissue as possible in the allotted time.
Coagulation	I could coagulate sufficient tissue.
Cutting ability	I could cut through the collagen fibres effortlessly.
Precision	I was able to fully control the laser energy application process at the given setting. I found that the procedure went smoothly.

Table 3: Formed pocket sizes in square centimeters for each laser setting and laser device. E_{SP}: energy of an optical pulse in Joule, f: pulse frequency in Hertz, τ: duration of an optical pulse in milliseconds, P: average power in Watt.

**Laser device**	**E_{SP} [J]**	**f [Hz]**	***P* [W]**	**τ [ms]**	**Tissue pocket size [cm²]**
**HP-Ho:YAG**	3	10	30	0.8	4.5
	3	25	75	0.8	8
	4.5	22.3	100	0.15	31.5
	4.5	22.3	100	0.8	2.25
**LP-Ho:YAG**	2.5	12	30	0.34	4.5
	2.5	12	30	0.41	2
	3	10	30	0.44	2
	3.5	10	35	0.45	6
**TFL**	0.2	200	40	0.4	0.25
	3	10	30	6	5.25
	3.5	10	35	7	5.25
	4	10	40	8	12
	4.5	8.9	40	9	1
	6	6.7	40	12	0.25
**p-Tm:YAG**	1.2	100	12	0.40	3.75
	2	50	100	0.6	8
	3	10	30	0.79	3
	3	25	75	0.86	15
