# Supplementary figures and images for: Experimental ex-vivo performance study comparing a novel, pulsed thulium solid-state laser, chopped thulium fibre laser, low and high-power holmium:YAG laser for endoscopic enucleation of the prostate
Source: World J Urol. 2021 Sep 3;40(2):601–6. doi: 10.1007/s00345-021-03825-z (PMC8921029; doi:10.1007/s00345-021-03825-z)

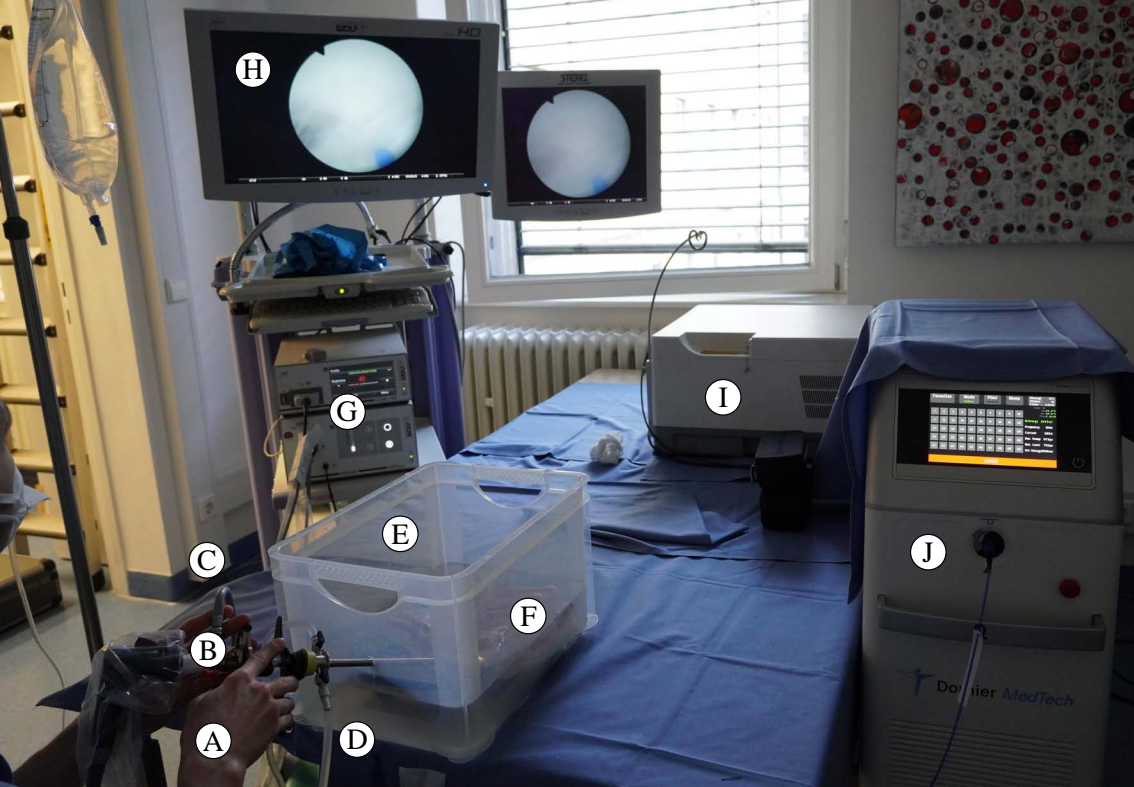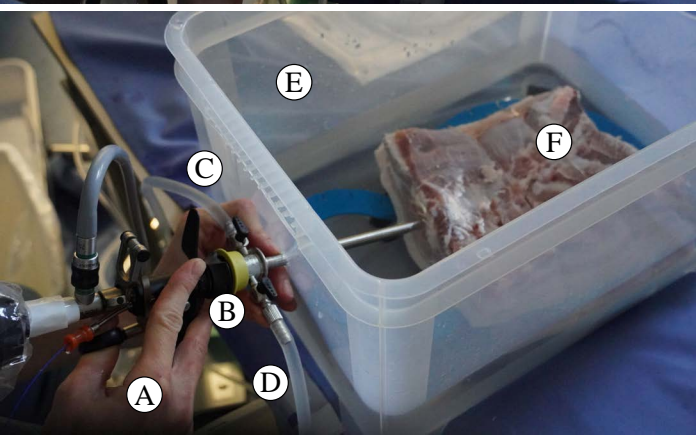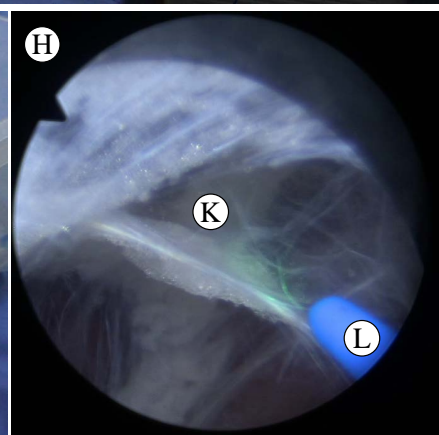

Supplement: Supplementary file 2 — Supplementary file2 Online Resource 2 Legend. Fig.1 Experimental setup: A) surgeon’s hands holding the endoscope, B) endoscope, C) and D) inflow and outflow of irrigation, E) box, F) porcine belly, G) image and light transmission system of endoscope, H) endoscopic image of the porcine belly, I) TFL, J) p-Tm:YAG, K) collagen fibres, L) laser fibre tip. (PDF 224 KB) [file 345_2021_3825_MOESM2_ESM.pdf]

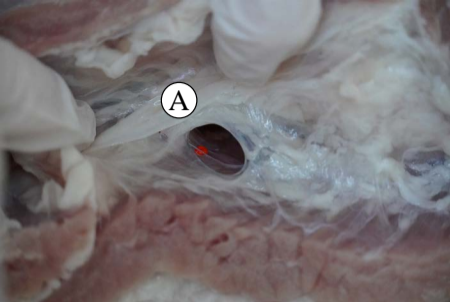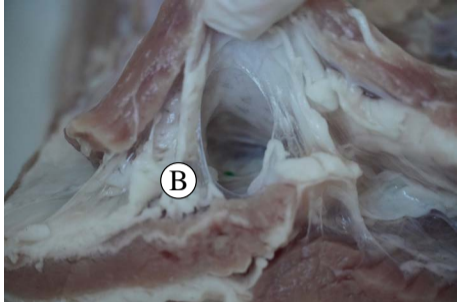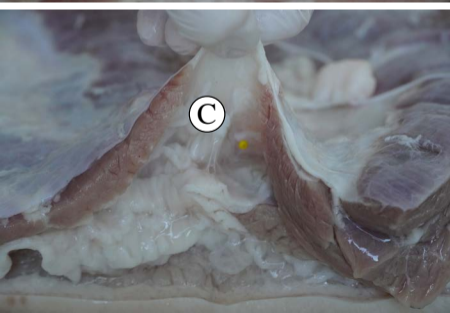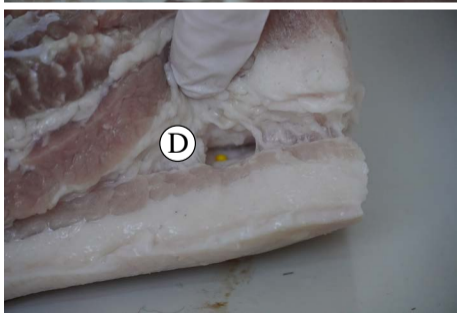

Supplement: Supplementary file 3 — Supplementary file3 Online Resource 3 Legend. Fig.2 Tissue pockets created by different laser devices applying the same laser setting (3 J, 10 Hz): A): LP-Ho:YAG (2 cm²), B): TFL (5.25 cm²), C): HP-Ho:YAG (4.5 cm²), D): p-Tm:YAG (3 cm²) (PDF 101 KB) [file 345_2021_3825_MOESM3_ESM.pdf]

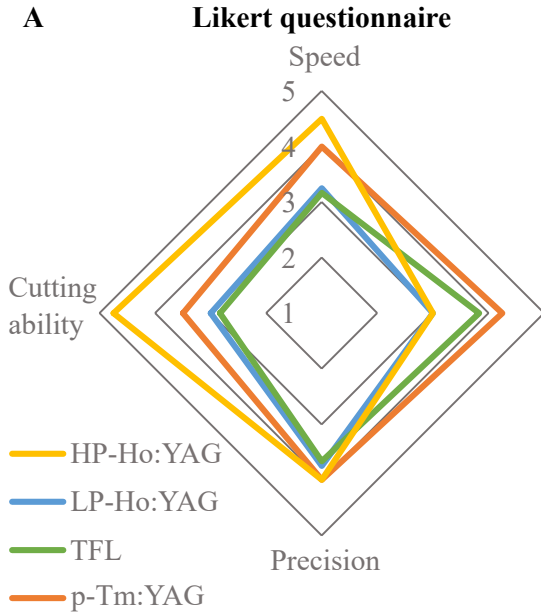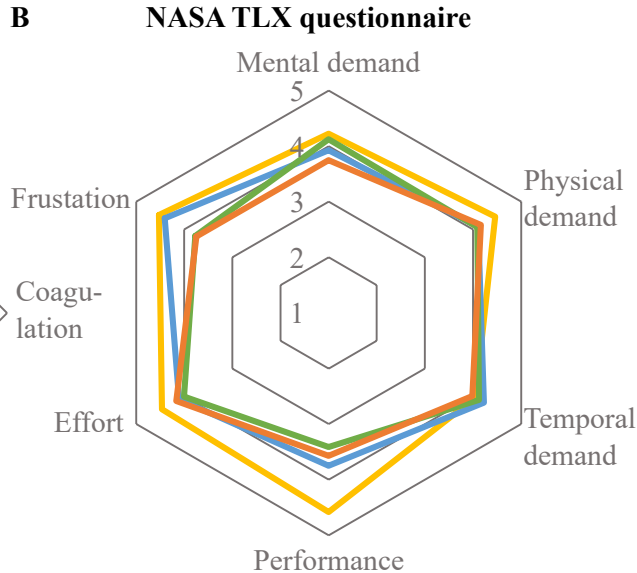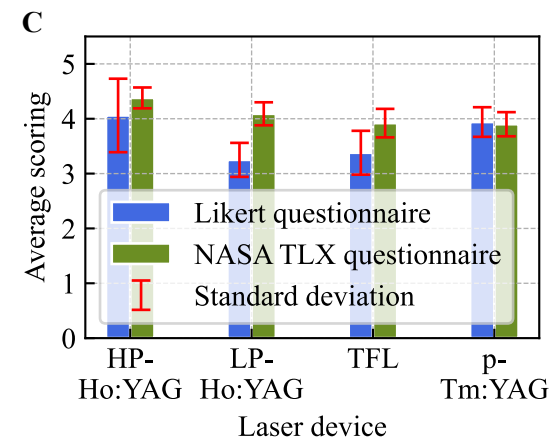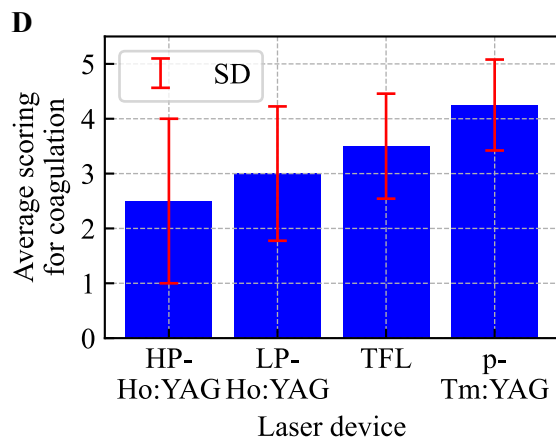

Supplement: Supplementary file 4 — Supplementary file4 Online Resource 4 Legend Fig.3 A and B: Laser devices’ mean scores on the Likert and NASA-TLX questionnaires illustrated as net diagram; C: Each laser device’s average score on the Likert and NASA-TLX questionnaires; D: Relation between average scores of coagulation performance and laser devices assessed by an independent, non-urologist observer. The red error bars indicate the standard deviation (SD) (PDF 138 KB) [file 345_2021_3825_MOESM4_ESM.pdf]
